# Perturbations of whole-brain model reveal critical areas related to relapse of early psychosis

**DOI:** 10.1162/NETN.a.502

**Published:** 2026-01-08

**Authors:** Iraïs Garcés de Marcilla Lappin, Ludovica Mana, Yasser Aleman-Gomez, Luis Alameda, Alessandra Solida, Raoul Jenni, Philipp S. Baumann, Paul Klauser, Philippe Conus, Morten Kringelbach, Patric Hagmann, Gustavo Deco, Yonatan Sanz Perl

**Affiliations:** Center for Brain and Cognition, Computational Neuroscience Group, Universitat Pompeu Fabra, Barcelona, Spain; Lausanne University Hospital and University of Lausanne (CHUV-UNIL), Lausanne, Switzerland; Department of Psychosis Studies, Institute of Psychiatry, Psychology and Neuroscience, King’s College London, National Psychosis Unit, South London and Maudsley NHS Foundation Trust, London, UK; Service of General Psychiatry, Treatment and Early Intervention in Psychosis Program, Lausanne University Hospital (CHUV), Lausanne, Switzerland; Department of Psychiatry, Instituto de Investigación Sanitaria de Sevilla, IBiS, Hospital Universitario Virgen del Roco, Universidad de Sevilla, Sevilla, Spain; Center of Psychiatry of Neuchâtel (CNP), Neuchâtel, Switzerland; Center for Psychiatric Neuroscience, Department of Psychiatry, Lausanne University Hospital and the University of Lausanne, Lausanne, Switzerland; Service of General Psychiatry, Department of Psychiatry, Lausanne University Hospital and the University of Lausanne, Lausanne, Switzerland; Service of Child and Adolescent Psychiatry, Department of Psychiatry, Lausanne University Hospital and the University of Lausanne, Lausanne, Switzerland; Department of Psychiatry, University of Oxford, Oxford, UK; Center for Music in the Brain, Department of Clinical Medicine, Aarhus University, Aarhus, Denmark; Centre for Eudaimonia and Human Flourishing, University of Oxford, Oxford, UK; Institució Catalana de la Recerca i Estudis Avancats (ICREA), Barcelona, Spain; National Scientific and Technical Research Council (CONICET), Buenos Aires, Argentina

**Keywords:** Psychosis, Psychosis relapsing, Perturbations, Variational autoencoder, Whole-brain dynamics, Neural network classifiers

## Abstract

Overcoming an initial psychotic episode does not always lead to recovery; relapses and subsequent psychotic episodes may happen afterward. Even if the characterization of psychotic disorders can be related to alterations in brain connectivity, clear identification of the brain areas for relapse is missing. Here, we leverage on whole-brain modeling linking anatomical structural information with functional activity as measured by MRI in 196 participants. Patients were classified into Stage II (first episode), IIIa (incomplete remission), IIIb (remission followed by one relapse), and IIIc (remission followed by several relapses), depending on the course of psychosis up to the time of the brain scan. From these data, a low-dimensional manifold reduction of the brain dynamics was obtained using deep learning variational autoencoders in which the different stages are represented, and a classification model can be trained to distinguish them. Then, a dimensionality analysis was performed to find the optimal dimension that allows the distinction between first episode and relapsing cases with high accuracy. Finally, perturbations were introduced in the model to reveal the brain regions associated with the absence of relapse, which could help predict which brain regions to target during therapy and assist the treatment of patients suffering from psychotic disorders.

## Author Summary

The evolution of the psychotic patient can be difficult to predict, as subsequent episodes may arise. Therefore, even if the characterization of psychotic disorders can be related to alterations of structural and functional brain connectivity, a clear cause of these relapses has not been found yet. In this article, we develop a classification strategy based on whole-brain modeling linked with anatomical structures and brain activity. From the provided data, a low-dimensional space is created where the different psychotic stages, regarding the presence or lack of relapses is represented, and a classification model can be used to distinguish them with high levels of accuracy. Afterward, perturbations were introduced in the model to reveal the brain regions associated with the absence of relapse.

## INTRODUCTION

Psychosis can be defined as the loss of capacity to distinguish between what is real and what is not. The World Health Organization characterizes it as the presence of hallucinations and/or delusions, which are associated with an impaired perception of reality, and could result in the incapacity to meet ordinary demands of life (Bartholomeusz et al., 2017). Furthermore, in the Diagnostic and Statistical Manual of Mental Disorders, Fifth Edition, psychotic disorders also present other symptoms such as disorganized speech or/and behavior, catatonia (motor anomalies) or negative symptoms (which refer to a state of reduced emotional expression, decreased motivation, inability to feel pleasure and reduced spontaneous speech; Griswold, Del Regno, & Berger, 2015; World Health Organization, 1992). Psychosis is also a feature of multiple psychiatric or neurocognitive disorders, such as depressive and bipolar disorders, or that can be present in patients already suffering from dementia or Parkinson’s disease (Bartholomeusz et al., 2017). All of this means that the global prevalence of psychosis is around 3% (Griswold et al., 2015; McCleery & Nuechterlein, 2019).

Moreover, the etiology behind psychosis is still undetermined (McCleery & Nuechterlein, 2019). Despite the well-established interactions between genetic and environmental risk factors (van Os, Rutten, & Poulton, 2008), there is still no clear specific neurobiological marker that could help the precise diagnosis and/or aid in the prediction of the evolution of the disease. However, some associations have been observed between psychotic disorders and some alterations of some brain structures, their function, and connectivity, in addition to alterations in some neurochemical functions of the brain (Bartholomeusz et al., 2017; Fusar-Poli, Radua, McGuire, & Borgwardt, 2012). It is well established that gray matter volumes are decreased in patients suffering from a psychotic disorder when compared with healthy controls (HCs). For instance, this phenomenon has been observed as reductions in the right temporal and left anterior cingulate, cerebellar regions, and the insula (Fusar-Poli et al., 2012). Moreover, with every relapse, it has been observed that more severe cognitive deterioration occurs and the recovery possibilities decrease (Sullivan et al., 2017), and the risk of developing persistent psychotic symptoms increases (Alvarez-Jiménez, Parker, Hetrick, McGorry, & Gleeson, 2011).

Advances in brain imaging and its analysis allow us to map the neural network anatomy and the dynamics of its activity at different scales of the brain (Fornito, Bullmore, & Zalesky, 2017). In particular, at the whole-brain macro scale in which we focus, the brain can be conceptualized as an interconnected system, and this allows us to model the circuit-level dysfunctions that underpin psychiatric disorders (Deco & Kringelbach, 2014; Fornito et al., 2017). These developments could be the base of understanding the psychopathology of these conditions in new ways, as models generated through connectomes can allow us to identify essential interactions between different brain areas to comprehend these conditions and their evolution (Breakspear, 2017; Deco & Kringelbach, 2014; Griffa, Baumann, Thiran, & Hagmann, 2013; Kringelbach & Deco, 2020). Computational whole-brain models, based on functional connectivity (FC) and structural information, have been used to determine physical and biological phenomenon, such as global states of consciousness (Perl et al., 2023), neurodegeneration (Sanz Perl et al., 2023), or cognition (Deco et al., 2023). Focusing on psychotic disorders, other investigations also have used whole-brain modeling approaches to investigate the alterations in brain activity in terms of network wiring (Fornito et al., 2017), global signal (Yang et al., 2014), and brain dynamics (Mana et al., 2023), or to characterize those brain areas that could be more impaired in patients with schizophrenia (Griffa et al., 2015). Even more, during the last years, whole-brain computational models have been providing a promising avenue to investigate the response to external interventions for different conditions, not only in terms of prediction but also in terms of the interactions that underlies the brain states transitions (Deco & Kringelbach, 2014). In some circumstances, these models can be used to exhaustively generate in silico interventions, providing useful insights for in vivo clinical applications, which are typically restricted to a small number of options due to experimental and ethical constraints (Mana et al., 2023; Perl et al., 2023; Sanz Perl et al., 2023; Vohryzek et al., 2023).

Traditionally, the discovery of resting-state networks (RSNs) has greatly influenced the investigation of brain functioning, changing the focus from local regions to the small number of global brain networks. Recent studies have demonstrated that simplifying complex brain recordings makes them more useful for studying brain function (Misic, 2024). For example, in a recent work, Lee et al. (2024) have used and validated a methodology to quantify changes in whole-brain co-activation patterns derived from resting-state functional magnetic resonance imaging (fMRI) (Lee et al., 2024). Their analysis allows to find a low-dimensional space representation that simultaneously captures patterns that generalized over time within individuals and patterns that vary over time, giving a proper distinction of behaviors in a less computationally exhausting and more efficient space. However, the optimal of dimensions to describe brain function is an open neuroscientific question (Lee et al., 2024). Here, we ask what is the optimal low-dimensional representation that better captures the alterations of brain function in individuals with psychosis. In other words, how much complexity do we need to distinguish between psychotic stages characterized by the number of relapses untill the time of the scan? We propose a data-driven approach to reveal these dimensions by combining machine learning, data augmentation, and whole-brain computational modeling to generate a low-dimensional manifold in which the brain dynamics of psychotic patients is embedded. Importantly, we then leverage this methodology to provide insights about those brain areas that can be related to the evolution of the psychotic condition. To do so, we built whole-brain models fitted to different stages of psychosis that allow us to train a classifier that distinguished between them. Importantly, the psychotic stages are determined by the number of the psychotic episode of each subject until the time of the scanning. Then, we trained variational autoencoders (VAEs) to find the optimal dimension of a low-dimensional space for the classification of the different psychotic classifications, which allowed us to extract the relevant features for their optimal classification. Afterward, we introduced external perturbations in each of the defined brain areas in the dynamic models of the subjects and study the possible reclassification those perturbations could generate from the original classification to another. The creation of the previous low-dimensional space gave us a domain in which we were able to reclassify these changes in the classification and observe a possible relation to anatomical structures and the connectivity between them. Specifically, we hypothesize that this approach allows for the exploration of perturbations on each brain area to find which nodes may be associated with the level of relapse of the psychotic symptomatology.

## RESULTS

### Methodological Overview

**Figure 1.**
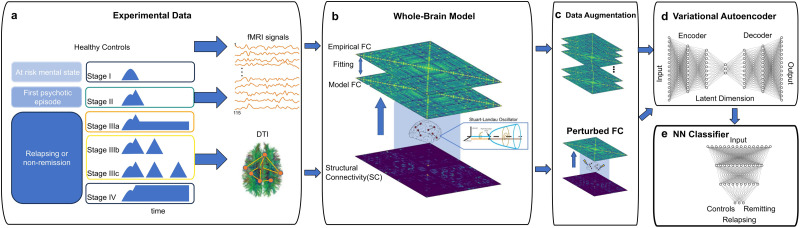
Methodological overview. From left to right: (A) Patients with schizophrenia were stratified into six clinical stages based on McGorry et al. (2014), reflecting the most advanced condition at the time of brain imaging (*x*-axis representing the time dimension, while blue areas reflected in the *y*-axis represents the strength of the psychotic episodes that characterise the evolution of the described psychotic stages). (B) A whole-brain model was fitted using DTI and fMRI data to simulate resting-state brain activity for three conditions: controls, nonrelapsing (II), and relapsing patients (IIIb and IIIc). The model incorporates anatomical connectivity (SC) and local dynamics governed by Stuart–Landau oscillators, with bifurcation parameter (A) and global coupling parameter (G) tuned to fit FC from the empirical data. (C) The tuned model was used to generate surrogate FC matrices for each condition and to perform in silico perturbations to test state transitions. (D) A variational autoencoder (VAE) was trained on the augmented FC data to produce a low-dimensional latent space representation of brain dynamics. (E) A neural network classifier was trained on this latent space to distinguish clinical conditions and to evaluate whether in silico perturbations could induce transitions between stages.

The methodological procedure implemented in this work is depicted in Figure 1 and is expanded in detail in the Materials and Methods section. In brief, we analyzed a neuroimaging dataset of patients with schizophrenia, categorized into distinct clinical stages based on a consensus evaluation following the staging model proposed by McGorry et al. (2014). Each patient’s stage reflected the most advanced condition present at the time of brain imaging. The classification comprised six groups: (a) Stage I included individuals at risk for psychosis but without definitive symptoms; (b) Stage II comprised patients in the early phase of the illness, including first-episode psychosis; (c) Stage IIIa involved patients who had not fully recovered 12 months after the initial episode; (d) Stage IIIb referred to those who experienced a single relapse following full remission; (e) Stage IIIc included individuals with multiple relapses separated by periods of full remission; and (f) Stage IV involved patients with no remission 1 year after the first episode (Figure 1, first column). We first implemented a whole-brain model to simulate brain activity in schizophrenia patients across different considered conditions (controls, nonrelapsing [II], and relapsing patients [IIIa and IIIb]) using diffusion tensor imaging (DTI) and fMRI data. This model captures the patterns underlying resting-state brain dynamics (Deco & Kringelbach, 2014). Specifically, it integrates anatomical connectivity with local neural dynamics to fit empirical neuroimaging data (Breakspear, 2017; Deco & Kringelbach, 2014; Kringelbach & Deco, 2020). Local dynamics are governed by nonlinear Stuart–Landau oscillators, which undergo a Hopf bifurcation, enabling transitions between oscillatory and noiselike behavior. Model fitting involved tuning the bifurcation parameter (a) for each brain region and a global coupling parameter (G), which scales the strength of anatomical connections (Figure 1, second column). After tuning, the model served two purposes: (a) as a data augmentation tool to generate surrogate FC matrices for each condition and (b) to perform in silico perturbations of whole-brain dynamics to test whether external inputs could induce transitions between clinical states (Figure 1, third column). We then used the augmented FC datasets to train a VAE, generating low-dimensional representations of brain dynamics (Figure 1, top last column). Finally, a neural network classifier was trained on these latent representations to distinguish clinical conditions. This classifier was then used to assess whether in silico perturbations induced transitions between stages (Figure 1, bottom last column).

### Fitting Whole-Brain Model of Psychosis Stages

**Figure 2.**
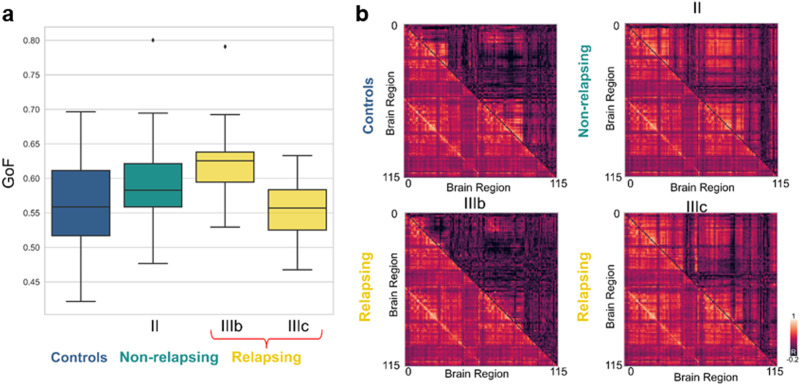
Fitting of the model for each psychotic stage: (A) Resulting of the distribution of the GoF across the training and fitting of the whole brain each of the different studied psychotic stages. (B) Comparison between the empirical (lower triangular) and simulated (upper triangular) FC matrices obtained for one optimization procedure for each studied psychotic stage.

The results of model fitting are shown in Figure 2. We quantified the level of a whole-model fit by computing the Pearson’s correlation between the upper triangular part of the simulated and empirical FC matrices. The Goodness of Fit (GoF) of the model is displayed in box plots across 20 iterations of the full genetic algorithm optimization procedure for HCs, psychotic Stages II (nonrelapsing), IIIb, and IIIc (relapsing) (Figure 2a). We noticed that in the four cases, the level of fitting is comparable, which is important to further investigate the model response to the perturbations. Figure 2b displays the simulated and modeled FC for one repetition of the optimization procedure for all the conditions. Importantly, the level of fitting we obtained is comparable to that achieved in previous works using the same model and a similar optimization procedure for other conditions, such as sleep (Ipiña et al., 2020), disorders of consciousness (Perl et al., 2023), and Alzheimer’s disease (Sanz Perl et al. (2023). However, we acknowledge that recent optimization procedures have been developed to fit fMRI empirical data by computing effective connectivity beyond functional brain dynamics (Schirner, Deco, & Ritter, 2023). These approaches infer connections between brain regions based on the FC and as consequence yield higher FC fitting values. However, leveraging that we used the structural connectivity (SC) of the same cohort of patients, we decided to fix the connectivity unaltered.

Following previous works the fitting procedure can be used to use the model parameters as biomarkers to distinguish and characterize different brain conditions using different models (Durstewitz, 2017; Frässle et al., 2017), we assessed whether our model parameters extracted for each psychotic conditions may be informative to distinguish between those states. To do so, we trained a neural network classifier (fully connected feedforward neural network [FCN]) using the 16 model parameters, meaning a and G for each RSN obtained for each of the three conditions (controls, relapsing, and nonrelapsing patients). We repeated 100 times, and we found that the classification accuracy was persistently lower than 0.5. Despite this accuracy being above chance, it is relatively low, thus we propose to investigate how low-dimensional representations of psychotic whole-brain activity could help to improve the classifications of these condition.

To further investigate whether the dynamical system data augmentation procedure provides useful information for distinguishing between the three classes, we trained two t-SNE algorithms: one using the empirical FCs and the other using the simulated FCs. The goal is to visualize the high-dimensional data in a two-dimensional space to examine how well the three classes are separated when empirical or simulated data are considered. We found that the two-dimensional representation of empirical FCs for the three classes is less separate than the one obtained using simulated FC (mean class distance for empirical FC = 6.4346, mean class distance for simulated FC = 27.4232) (Supporting Information Figure S2 and Supporting Information Figure S3).

### Low-Dimensional Representation of Diseases: Optimal Dimensionality

**Figure 3.**
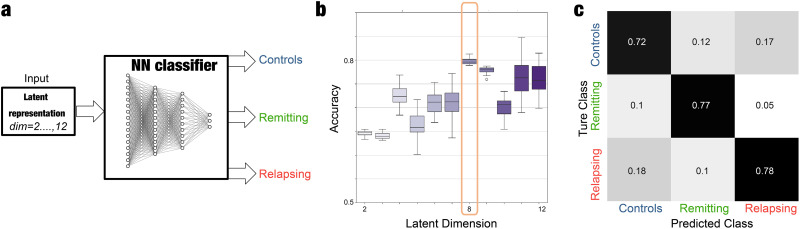
Optimal low-dimensional representation to classify the nonrelapsing and relapsing psychotic stages. (A) Scheme of the neural network (FCN) classifier. (B) The performance of the neural network classifier trained to distinguish between the three classes as a function of the dimension of the latent space. The accuracy increases from Dimension 2 up to 8, when it seems to reach an elbow and then the values remain constant. We considered 8 as the optimal dimension of the latent space representation. (C) The confusion matrix for an optimal classification on the 8D latent space.

To analyze the power that the different selected dimensions represented in terms of aiding in the classification of the psychotic state, we have based our analysis on a FCN architecture, the scheme of which can be seen in Figure 3a. The input of the network is the projections in each latent coordinate of the different encoded FC matrices for the different selected dimensions, while the output is one of the three considered classes: HCs, nonrelapsing, and relapsing patients.

By performing fivefold cross-validation and repeating the procedure 100 times per dimension, we demonstrated that the selected hyperparameters are consistent in terms of performance. We also assessed the statistical significance of the accuracy values by training and evaluating 100 FCNs classifiers using the same features as those in Figure 3, but with class labels randomly scrambled for each dimension (Supporting Information Figure S4 and Supporting Information Figure S5). Additionally, we have included an extra computation using a new test set to provide further evidence supporting our hyperparameter selection (Supporting Information Figure S6). To achieve this, we leveraged the augmented data generated from whole-brain models for each condition to create a test set that was unseen during the training of the 100 FCN classifiers. The results obtained for this analysis are reported in Figure 3b where we observed that the performance in the classification increases as more dimensions are added until reaching a classification accuracy of 0.8. Here, we see that from Dimension 2 until 7, the accuracy gets higher for each new dimension that is defined in the creation of each VAE model. However, once Dimension 8 is reached, the precision of this classification does not increase in a relevant trend, leading us to believe that by this methodology, higher accuracy values are difficult to reach as we continue increasing the number of dimensions of the latent space. Furthermore, this behavior indicates that the optimal classification of the different studied psychotic stages can be performed in an eight-dimensional space, as seen in the confusion matrix obtained with a FCN classifier in the optimal 8D latent space is displayed in Figure 3c.

Interestingly, the accuracy of the classifier using the 8D latent representation is significantly higher compared with that achieved using the model parameters, suggesting that this low-dimensional representation could be informative. To further assess the relevance of our methodological approach, we trained 100 FCNs to distinguish between the three classes using empirical FCs, obtaining the reported accuracy 0.67 ± 0.07. We also trained 100 FCNs using the 8D representation obtained by encoding the empirical FCs, achieving an accuracy of 0.66 ± 0.6. Importantly, both cases showed significantly lower accuracy levels compared with those obtained by encoding in 8D the simulated FC (*p*_*val*_ < 0.05 Kolmogorov–Smirnov test in both comparison) (Supporting Information Figure S1). Remarkably, this approach is based on encoding and classifying the three classes based on the corresponding FCs. However, while we are creating whole-brain models for each condition, we are able to investigate the impact of each brain regions by performing in silico perturbations and addressing how prone is to induce transitions from psychotic condition toward a healthy state.

### In Silico Perturbation Induce Transitions

**Figure 4.**
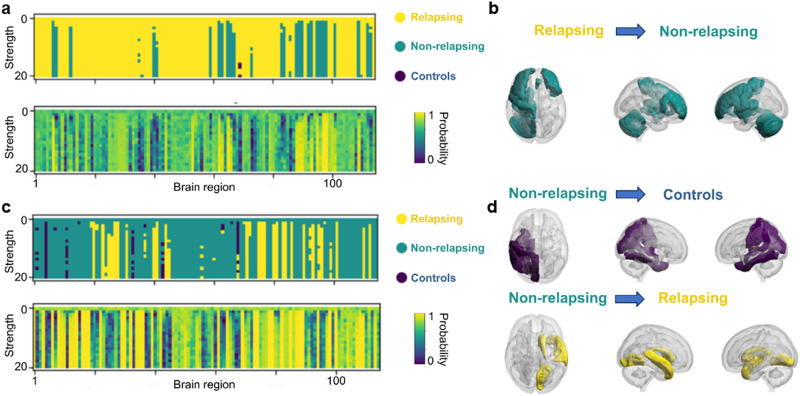
In silico perturbations of the different models: (A) Resulting classification (and the certainty of each classification) of the FC matrices after the perturbation of an original relapsing case. The *x*-axis represents the perturbed region (node j, from 1 to 115), the *y*-axis represents the strength of the external periodic forcing (the amplitude described in Equation 5), and the color scale indicates the reclassified condition (upper row) and the probability of reclassification (lower row); (B) Perturbability map: representation of the brain areas whose perturbation led to changes in the global dynamics associated with a reclassification from relapsing to nonrelapsing psychotic stage. (C) Resulting classification (and the certainty for each classification) of the FC matrices after the perturbation of an original nonrelapsing case, distributed by node and amplitude of said perturbation. (D) Perturbability maps: representation of the brain areas whose perturbation led to changes in the global dynamics associated with a reclassification from nonrelapsing to relapsing psychotic stage and nonrelapsing to healthy control.

After finding the ideal low dimensional space for the classification of the different defined psychotic stages, we simulated a perturbation by the addition of an external periodic force to the optimal model corresponding to relapsing and nonrelapsing psychotic stages. We explored different model perturbations by varying the strength of the force and stimulated individual brain regions (see Escrichs et al., 2022, for details of the perturbation approach). This procedure allowed us to obtain a series of perturbed FC matrices as a function of these two variables. We quantified the responsiveness to the external stimulus by evaluating whether the classification obtained in the 8D latent space remained as the unperturbed state or changed. In Figure 4a, upper row, we can observe how the classification of our synthetic FC matrices change when different areas of our parcellation are perturbed at different intensities. Those perturbations are introduced to see if a reclassification of the original psychotic stage can be accomplished by an external stimulation and provoke a change in the dynamics of a relapsing patient that leads to its reclassification by the FCN classifier. In the lower row, we show the probability of the associated changes as a measure of the classification confidence. The brain regions that induce changes in the brain dynamics that yield a reclassification with a confidence level higher than a value of 0.6 from relapsing toward nonrelapsing stage are shown in Figure 4b. This visualization highlights the relation between the perturbations applied to different regions of the left hemisphere and changes in the classification. The confidence level for this classification was obtained directly from the output of the classification model, indicating how certain the model was about its decision. Specifically, for each in silico perturbation and condition, we obtained the probability of being classified into each of the three original conditions. The highest probability was then compared with a predefined threshold of 0.6 to assess whether the classification could be considered reliable. Notice that the threshold of 0.6 was selected because the model was tasked with distinguishing among three equally likely categories. Since the combined confidence across all classes must equal 1, and the random chance of selecting the correct class is about 0.3, the threshold was set to twice that value to ensure sufficient confidence in the model’s prediction.

Then, we repeated the same procedure to evaluate the response of the nonrelapsing stage. In Figure 4c, it is displayed the reclassification in the 8D latent space after the perturbation as a function of the perturbed brain region and the forcing strength (upper row) and the probability of that reclassification (lower row). The regions that induce reclassification with high confidence are shown in Figure 4d. We can observe how inducing changes in the dynamics of the specific areas (highlighted) in, primarily, the left hemisphere produces changes in the classification from the nonrelapsing category to the control one, and from the relapsing category to the nonrelapsing one. On the other side, changes in the dynamics of the specific areas in the right hemisphere induce a shift in the classification from nonrelapsing dynamics to relapsing ones.

## DISCUSSION

This study is based on the use of a phenomenological model derived from fMRI dynamics, which are classified according to the stage of psychosis in nonrelapsing or relapsing psychotic patients, as well as HCs. We accomplished this by training a VAE model to generate a low-dimensional latent space in which accurate classification of these different psychotic stages could be achieved. Specifically, we found that the VAE model generating a low-dimensional space of eight dimensions provided the optimal information for classification. Furthermore, the proposed framework was compared against other training conditions for the distinction of the defined psychotic stages (i.e. using the bifurcation parameters for the classification, training the VAE with the empirical FC matrices and t-SNE representations) to assess the validity of our approach. These analyses are reported in the Supporting Information, where it can be observed that the presented approach outperforms other analyses.

Moreover, the use of this deep learning architecture enabled us to explore how FC matrices behaved after specific perturbations were applied to the original dynamics for each defined psychotic class. We were able to identify the key regions that, once perturbed, were associated with changes in global dynamics for the reclassification into another of the defined stages in our synthetic dataset, as we observed that specific node perturbations of the defined psychosis stages could lead to a reclassification into another category with enough confidence to be considered significant.

In particular, the crucial areas related to a change from a relapsing to a nonrelapsing state are mostly associated with structures in the cortex such as some frontal and left temporal areas, a subregion of the cingulate and the left insula. Outside the cortex, the hippocampal tail and the left cerebellum are also associated with this reclassification. Interestingly, alterations in the frontal and temporal areas have been related to higher vulnerability to psychosis and full onset of the disease (Fusar-Poli et al., 2012; Niznikiewicz, 2019), while worse prognosis have been associated with alterations in the cingulate and cerebellar areas (Fusar-Poli et al., 2012; Niznikiewicz, 2019). Furthermore, perturbations in the node corresponding to the left amygdala and thalamus have been associated with changes in the condition in both nonrelapsing and relapsing cases during our simulations, leading nonrelapsing cases to be reclassified as control cases, and relapsing cases to be reclassified as nonrelapsing. This relates to what is found in the literature, where deterioration in connectivity in the limbic system is associated with chronic stages of psychosis or higher risk of developing a psychotic disorder (Niznikiewicz, 2019). Therefore, as our perturbations model a stimulation in the described areas, which would enhance the connectivity in the limbic regions, the reclassification into nonpsychotic stages is observed.

Regarding the case of transitions from nonrelapsing stages, we also found crucial brain regions in which perturbations lead to a reclassification into the control category. Some of those areas are located in the cortex in the left temporal lobe and near the hippocampus, and as seen in the Introduction section, the temporal gyrus, encompassed in the highlighted areas in our analysis, is a structure in which the degradation of its gray matter has been previously associated with psychosis through the literature (Fusar-Poli et al., 2012). Therefore, a proper stimulation, as the one simulated in our perturbation analysis, could enhance the connectivity between those areas and theoretically lead to control-like dynamics. Moreover, perturbations of different regions showed that changes from nonrelapsing to relapsing dynamics can be induced in our model. These changes were associated to nodes in the right temporal regions, leading from an original nonrelapsing case to a reclassification into the relapsing category after the perturbation.

The relation of the results to what has been observed in the literature, as seen in this section, shows us that inducing perturbations in the described models by disturbing the original dynamics leads to changes in the classification that can be related to current hypothesis between the stages in the psychotic disorder and certain brain areas. The changes in the classification of the synthetic subjects after the perturbation of different areas can be understood as possible changes in the dynamics of the connections of those classes, and we can relate them to anatomical structures previously associated to the studied condition. This approach shows that by the use of whole brain modeling we can find similar areas than the ones related to psychosis by other methodologies. And as proposed in Sanz Perl et al. (2021), where they use a similar approach to distinguish between patients at different levels of consciousness, this model could be adapted to predict the optimal brain regions to target during therapy and assist the treatment of those patients suffering from a psychotic disorder.

Regarding the limitations of this work, we need to take into account that the dataset originally used to generate the synthetic data comes from patients between 20 and 30 years old, with higher representation of male participants than female ones, and from the same hospital in Switzerland (Griffa et al., 2019). All these factors lead to bias in the creation of the synthetic dataset, meaning that the obtained results are more representative for patients with the same age range, biological sex, and from the same region than for others with different demographics.

Also, the accuracy of the prediction can be affected by the number of cases on which it was trained, as the VAEs and the FCN models could have been trained with a larger dataset; however, due to computational limitations, this was not feasible. Moreover, this also conditioned that more repetitions of the classification of the perturbed results could not be performed. These would have been useful to see possible mistakes in the classification of the perturbed FC matrices; however, due to the time these calculations took, it was not possible to perform such confirmation. Therefore, further validation with bigger datasets and more repetitions after the perturbation could be used to further validate these results.

Furthermore, regarding the classification of the dataset, we must consider that the classification of the patients in the different psychotic stages was done regarding their condition at the time of the scan, meaning that is possible that some patients labeled as nonrelapsing had a relapse after the information was extracted, which would lead to a change in their classification that was not considered here. This ongoing definition of the disease limits our understanding of the results as we cannot affirm that all these patients labeled as nonrelapsing did, in fact, had a complete remission of the condition. We can only affirm that the situations present here are the ones observed when the scans were taken. Moreover, the limits of the individual fitting of this approach to personalized cases still needs to be tested, as average SC matrices are used for each of the groups instead of the individual SC, as their use during training would lead to lower levels of generalization of the results.

Finally, to sum up, we can conclude that the framework combining machine learning and whole-brain modeling proposed in previous works (Perl et al., 2023; Sanz Perl et al., 2023) can be adapted for the analysis and understanding of the psychotic condition. It has to be highlighted that by exploring the dimensionality of the latent representation, we demonstrated that an eight-dimensional latent space provides an optimal classification of psychotic stages and allows us to generate classifiers to distinguish between the different psychotic stages regarding the course of the illness with high accuracies. Furthermore, the proposed model was compared against other training conditions for the disctiction of the defined psychotic stages (i.e., using the bifurcation parameters for the classification, training the VAE with the empirical FC matrices, t-SNE representations …) to assesst the validity of our approach. These analysis are reported in the Supporting Information, where it can be observed that the reported approach outperforms the other analysis. Moreover, we found some brain areas that could be related to changes in the psychotic condition and its relapsing during our perturbation of synthetic cases though our perturbation analysis.

## MATERIALS AND METHODS

### Participants

The data used for this work are derived from a dataset described in Griffa et al. (2019) and in Mana et al. (2025), including a total of 216 subjects: 88 early psychosis (EP) patients and 128 HCs. EP patients were recruited from the Treatment and Early Intervention in Psychosis Program (TIPP) of the Lausanne University Hospital, Switzerland (Baumann et al., 2013), meeting psychosis threshold (more details regarding the characteristics of the dataset are given in the references Griffa et al. [2019]; Mana et al. [2025]). All of the participants provided informed written consent for this study, and the procedure was approved by the Ethics Committee of Clinical Research of the Faculty of Biology and Medicine, University of Lausanne, Switzerland.

The patients were initially stratified into distinct groups (Figure 1) based on a consensus assessment by two experienced psychiatrists, according to the clinical staging model proposed by McGorry et al. (2014). The clinical stage was rated as the highest condition achieved at the time of brain imaging based on a consensus assessment proposed by McGorry et al. (2014). According to this classification patients were initially stratified into five distinct stages: (a) Stage I patients who had been defined as being at risk of developing a psychotic disorder, but showed no definitive symptomatology; (b) Stage II patients in the early stages of the disease, with a first episode psychosis (FEP) and not fully symptomatic at the time of the scan; (c) Stage IIIa patients with incomplete remission from Stage II at 12 months after observations; (d) Stage IIIb patients with a single relapse of psychosis after full remission; (e) Stage IIIc patients with two or more relapses after of psychosis and full remission between episodes; and (f) Stage IV patients with no remission from Stage II after 12 months.

For the purpose of this study, we have classified EP participants into two subgroups: relapsing patients and those without observed relapses by the time of the scan. The 37 patients from Stage II will be our FEP category (i.e., no relapse); however, as the clinical stage was defined as the highest stage at time of scanning, Stage II patients may have been scanned either close to the first episode or later during the program if the patient did not present a second psychotic episode. They all were rated as Stage II because no relapse was observed after the first episode up to the scanning date; however, it does not assure us they did not present other psychotic episodes after the scan. On the other hand, 22 patients from Stage IIIb and nine patients from Stage IIIc were grouped together and classified as the relapsing category, as the only difference between them is the number of relapses and our main goal is to distinguish between individuals who did not relapse from those who did. The remaining patients, classified in Stage I, Stage IIIa, and Stage IV, will not be considered for the analysis, as they present profiles out of the scope of this research. The final dataset included in this study consists, therefore, of 128 healthy control subjects and 68 psychosis patients.

### Parcellations

Regarding the gray matter parcellation process, from individual T1-weigthed (T1w) images, cortical surfaces were reconstructed via FreeSurfer stream (v6.01) and subcortical regions were parcellated. The hippocampal subfields and brainstem were segmented using methods proposed by Iglesias et al. (2015), while for thalamic nuclei segmentation, an atlas-based approach with Advanced Normalization Tools was followed. The resulting segmentations were combined with the FreeSurfer outputs to generate the resulting gray matter parcellation of 115 brain regions, which allowed the generation of individual connectomes by estimating a combination of the magnetization-prepared rapid acquisition gradient echo (MPRAGE) and the diffusion spectrum imaging (DSI) data, giving a final parcellation of 115 cortical and subcortical regions based on a modified version of the Desikan–Killiany atlas (Desikan et al., 2006) proposed in Mana et al. (2023).

More information about its acquisition and initial processing can be found in Griffa et al. (2019) and Mana et al. (2023, 2025).

### MRI Data Acquisition and SC Reconstruction

The connectivity information was extracted from a MRI scanning using a 3-Tesla Siemens system, which was used for acquiring single-participant data, that included a MPRAGE sequence (1 mm in-plane resolution, 1.2 mm slice thickness) and DSI (257 diffusion-weighted directions, 1 non-diffusion-weighted (*b*_0_) volume, with a maximum b-value of 8000 s/mm and a spatial resolution of 2.2 × 2.2 × 3 mm^3^). Imaging protocols were carefully aligned before and after the upgrade, and the same 32-channel head coil was consistently used.

The connectome for each participant was derived by integrating MPRAGE and DSI data. MPRAGE images were segmented into white matter, gray matter, and cerebrospinal fluid, and the gray matter was parcellated into 115 anatomical regions (see Parcellations section). DSI data were reconstructed to compute generalized fractional anisotropy and apparent diffusion coefficient maps and to perform deterministic streamline tractography. SC between brain regions was quantified by the number of streamlines connecting each pair, producing weighted, undirected networks with 115 nodes. Furthermore, the presence of streamlines is not the only defining characteristic conditioning the SC but also the size of the regions themselves. Taking this into account in this definition of the connectivity normalizes the number of streamlines and assures that a more accurate representation of the true connectivity can be obtained. Therefore, to compute the density connectomes, the number of streamlines connecting each pair of regions was divided by the geometric mean of their volumes, thus obtaining a density measure that indicates the relative strength of the connectivity that finally generated the SC matrix.

Additional information on MRI acquisition parameters, preprocessing steps, and connectome construction is provided in Griffa et al. (2019).

### fMRI Data Acquisition and Great Average FC Computation

fMRI data were acquired from two 3-Tesla scanners, the Magnetom TrioTim, and PRISMA from Siemens Medical Solutions, and the resting-state fMRIs were sequenced with a repetition time of 1920 ms and 3.3 mm isotropic voxels. The acquisition times for the MPRAGE-T1w, rs-fMRI sequences were approximately 7, 13, and 9 min. Image quality assessment, including visual inspection and quality control metrics, ensured dataset quality. More details can be found in Mana et al. (2025).

Image processing involved gray matter parcellation with skull-stripping using CAT12 and FreeSurfer for cortical surface reconstruction and subcortical parcellation creating a final subdivision of gray matter in 115 regions of interest. The fMRI preprocessing was conducted with fMRIPrep, including motion correction, co-registration with anatomical images, spatial normalization, slice-timing correction, smoothing, and nuisance signal regression. Neuroimaging procedures are described in full detail in Mana et al. (2025). Finally, fMRI signals were detrended and demeaned before band-pass filtering in the 0.04–0.07 Hz range (Ipiña et al., 2020). Previous works have shown that this band contains more reliable and functionally relevant information compared with other frequency bands, and to be less affected by noise (Ipiña et al., 2020). The individual FC matrix was defined as the matrix of Pearson correlations between the fMRI signals of all pairs of regions in the parcellation for each participant. Then, the FC for each class was obtained as the average over patients in each condition and healthy controls.

### Whole-Brain Model

Afterward, we implemented a whole-brain model of coupled nonlinear oscillators to fit fMRI recording of the studied cases based on previous works (Deco, Kringelbach, Jirsa, & Ritter, 2017; Ipiña et al., 2020; Sanz Perl, Escrichs, Tagliazucchi, Kringelbach, & Deco, 2022), as it has been demonstrated that these models allow us to study the dynamics of the brain with higher accuracy than raw fMRI data. The local dynamics (one per each brain region in the 115 cortical and subcortical used parcellation) is determined by the normal form of a Hopf bifurcation (Deco et al., 2017), which allows for changing the dynamics between noise and oscillations in the critical point to a stable fixed point in the phase space (Sanz Perl et al., 2022). Each node (brain region) was coupled by the anatomical SC matrix obtained from DTI measurements. The strength of the coupling can be scaled by a coupling parameter representing the global conductivity, *G*, while the bifurcation parameter was defined by *a*, which defines the dynamic behavior of each brain area. The model parameters were optimized to fit the simulated whole-brain FC matrices computed from the simulated time series to the empirical FC by means of the Pearson’s correlation. Moreover, the global dynamics of the brain network model results from the computation of mutual interactions of local node dynamics coupled through the underlying empirical anatomical SC matrix (*C*), which encodes the density of fibres between brain regions. The element *C*_*i*_*j* represents the connectivity between brain regions *i* and *j* within the considered brain parcellation. The dynamics of each node, describing transitions from asynchronous noisy behavior to oscillations is described by the following equations:

$$\frac{{dz}_{j}}{dt}=z\left[a_{j}+i\omega_{j}-|z_{j}^{2}|\right]+{\beta \eta }_{j}\left(t\right)$$ (1)

$$z_{j}=\rho_{j}e^{i\theta_{j}}=x_{j}+{iy}_{j},$$ (2)

where *η* represents an additive Gaussian noise with a standard deviation equal to *β* = 0.02. The supercritical Hopf bifurcation occurs at *a*_*j*_ = 0. As for lower values of *a*_*j*_, the local dynamics have a stable fixed point at *z*_*j*_ = 0, corresponding to a low activity asynchronous state, while for values of *a*_*j*_ > 0, there exists a stable limit cycle oscillation with frequency *f*_*j*_ = *ω*/2*π*.

Therefore, we can define the whole-brain dynamics as the following by separating the imaginary and real part of Equation 1:

$$\frac{{dx}_{j}}{dt}=\left[a_{j}-x_{j}^{2}-y_{j}^{2}\right]x_{j}-\omega_{j}y_{j}+G\sum_{i}C_{ij}\left(x_{i}-x_{j}\right){\beta \eta }_{j}\left(t\right)$$ (3)

$$\frac{{dy}_{j}}{dt}=\left[a_{j}-x_{j}^{2}-y_{j}^{2}\right]y_{j}+\omega_{j}x_{j}+G\sum_{i}C_{ij}\left(y_{i}-y_{j}\right){\beta \eta }_{j}\left(t\right)$$ (4)

Finally, Equation 3 and Equation 4 will be coupled using the common difference coupling for approximating their linear part, which allows sharing the connectivity information between the different nodes and model the desired brain dynamics.

Within this model, the intrinsic frequency *ω*_*n*_ of each node is in the 0.04- to 0.07-Hz band (*n* = 1, …, 115). The intrinsic frequencies of each brain region were empirically estimated from the data, as given by the averaged peak frequency of the narrowband BOLD signals across participant. Importantly, for each of the three considered conditions we obtained a set of intrinsic frequencies *ω*_*n*_ (Deco et al., 2017; Ipiña et al., 2020).

### Fitting of the Model

For the optimization procedure we gathered brain regions by their belonging to the RSN and an independent parameter (*δ*) was fitted for each RSN, then these parameters were combined to obtain the local bifurcation parameters (*a*) and coupling strength (*G*) of each node in the model (Ipiña et al., 2020). This procedure seeks to minimize the GoF defined as *GoF* = 1 − *corr*(*FC*_*empirical*_, *FC*_*simulated*_), and this aims to balance the sensitivity to absolute and relative difference between the FC matrices. Furthermore, using the RSN to group the different brain regions into their RSNs, we further simplify the complexity of our model, as we can encode the studied parameters for each brain area in a RSN into a single representation parameter that represents said network and describes their contribution to the local dynamics.

Moreover, we build a different model for each studied category: healthy controls, patients that have only experienced one psychotic episode until the time of the scan or nonrelapsing patients (Stage II), and relapsing patients with more than one psychotic episode until the time of the scan (Stages IIIb and IIIc) while considering the group average SC for each group, by averaging the value of each connection between pairs of nodes (i.e., edges). This was used as a control variable during this procedure to reduce the variability that could arise in our model by using the particular SC for each subject.

### Data Augmentation

We implemented a dynamical model data augmentation procedure to generate surrogate FC data for each condition (Perl, Pallavicini, et al., 2020) that we used to train a deep learning architecture known as VAE (Perl, Bocaccio, et al., 2020), giving us a final dataset of 3,000 subjects (1,000 subjects per class). The genetic algorithm used in this step was based in the minimization of the GoF while training the mathematical model. The first generation of this algorithm consisted of 20 individuals with random values and their corresponding GoF, then the one with the best fittings were chosen and used as input to generate new individuals, which again had their GoF measured and compared untill the stopping criterion was met (20 generations are reached or the average GoF across the generation is 10^−6^). The 20 final individuals of this process were saved to be used as starting individuals in a new round of the genetic algorithm to create the final balanced dataset. After this round of augmentation, each class consisted of 1,000 synthetic subjects.

### VAE and FCN Training

In brief, VAEs are deep neural networks with AE architecture (Kingma & Welling, 2013), which are trained to map inputs to probability distributions in a latent space by minimizing the error between the input and the output, which captures more of the data’s variability than classical autoencoder models.

The VAE architecture can be subdivided into three parts: the encoder network, the middle variational layer (with units corresponding to the latent space), and the decoder network. The encoder transforms the input into the latent space, which is of much lower dimension than the input and output layers, while the decoder mirrors the encoder to convert the values of the units in the latent space to the output space. Regarding the training of the VAE networks, a single training cycle was performed consisting of 10 training cycles and a 128 batch size without minibatches. After training, we investigated the optimal latent dimension by quantifying the accuracy by neural network classifiers (FCN) trained to discriminate between the three considered classes (controls, relapsing, and nonrelapsing patients) of synthetic FC information.

Then, 100 FCNs were trained with the latent space coordinates generated, for each subject, during the encoding process for each studied dimension of the latent space (from two to 12 dimensions) and their accuracy computed to understand the classification power of each dimension and the robustness of our findings. The training of 100 classifiers per dimension was decided after balancing the need of having a large enough number of trials that could encapsulate the randomness in each creation of a FCN and the time those computations took. We observed that 100 FCNs per class yielded consistent accuracy distributions for all studied dimensions. Consequently, we used these 100 classifiers to assess the statistical significance of the accuracy values in each dimension. To do so, we trained and evaluated a set of 100 new classifiers using the same features but scrambling the class labels. We found that for all the dimensions the actual classifiers were significantly higher than the scrambled (Supporting Information Figure S4 and Supporting Information Figure S5). Moreover, each of these classifiers was trained and tested individually with a five k-fold cross-validation strategy, where 100 synthetic cases where isolated for the training data and saved for testing of the performance of each trained network in an unseen dataset (Supporting Information Figure S6). Then, the remaining dataset went through an 80/20 percent split (where 80% of the set was used for training and 20% for validation), 30 training cycles, 32 batch size, and an Adam optimizer with a learning rate of 0.001 were used for training the classifiers. The defined architecture for the FCNs consisted of four sequentially linked dense layers of 64, 32, and 32 nodes, with the last layer having as many nodes as classes.

### In Silico Perturbations

Furthermore, we explored in silico external wave stimulation defined as periodic perturbation of different amplitudes delivered at the intrinsic frequency of each node. This is done by systematically applying these perturbations to all nodes and different intensities of the perturbation (or strengths) and computed the resulting FC matrices. The chosen perturbation has previously been used for similar purposes in previous works (Escrichs et al., 2022; Sanz Perl et al., 2021) and it has been used as a conceptual model of the effects of transcranial alternating current stimulation. It is defined as a wave simulation that incorporates an additive periodic term into the dynamics of each brain region, which generated new FC matrices that can be further analyzed. These perturbations are described by the following equation:

$$F_{0}\text{cos}\left(\omega_{0}t\right)$$ (5)

*F*_0*j*_ is defined as the forcing strength (amplitude of the perturbation in signal amplitude by time units) applied to each node *j*, which is explored in our analysis, and *ω*_0*j*_ is described as the intrinsic frequency of node *j*, computed from the BOLD signals for each condition. Note that the rationale for forcing each node at its intrinsic frequency is based on the idea that this maximizes the effect of the stimulation, as it corresponds to the node’s resonant frequency. This strategy was successfully tested in previous work allowing to induce transition between different brain states such as reduced states of consciousness toward wakefulness state (Ipiña et al., 2020; Sanz Perl et al., 2021, 2022).

Then, after the introduction of the described perturbations we encoded these perturbed FC into the low-dimensional latent space and reclassified them using the VAE and the neural network classifier respectively, which were previously trained with the unperturbed FC. And, finally, we categorized each perturbation in terms of its capability to generate a change in the classification compared with the unperturbed case.

## ACKNOWLEDGMENTS

Y.S.P. was supported by a number of sources: NEMESIS project (ref. 101071900) funded by the EU ERC Synergy Horizon Europe. G.D. was supported by a number of sources: NEMESIS project (ref. 101071900) funded by the EU ERC Synergy Horizon Europe; AGAUR research support grant (ref. 2021 SGR 00917) funded by the Department of Research and Universities of the Generalitat of Catalunya; project PID2022-136216NB-I00 financed by the MCIN/AEI/10.13039/501100011033/FEDER, UE, the Ministry of Science and Innovation, the State Research Agency and the European Regional Development Fund. P.K. was supported by a fellowship from the Adrian and Simone Frutiger Foundation. P.H. was financially supported by Swiss National Science Foundation Grant #320030–197787/1.

## SUPPORTING INFORMATION

Supporting information for this article is available at https://doi.org/10.1162/NETN.a.502.

## AUTHOR CONTRIBUTIONS

Iraïs Garcés de Marcilla Lappin: Conceptualization; Formal analysis; Methodology; Writing – original draft. Ludovica Mana: Writing – review & editing. Yasser Aleman-Gomez: Writing – review & editing. Luis Alameda: Writing – review & editing. Alessandra Solida: Writing – review & editing. Raoul Jenni: Writing – review & editing. Philipp S. Baumann: Writing – review & editing. Paul Klauser: Writing – review & editing. Philippe Conus: Writing – review & editing. Morten Kringelbach: Writing – review & editing. Patric Hagmann: Writing – review & editing. Gustavo Deco: Conceptualization; Data curation; Methodology; Project administration; Supervision; Writing – review & editing. Yonatan Sanz Perl: Conceptualization; Data curation; Formal analysis; Methodology; Project administration; Supervision; Writing – review & editing.

## FUNDING INFORMATION

Yonatan Sanz Perl, EU ERC Synergy Horizon Europe, Award ID: 101071900. Gustavo Deco, EU REC Synergy Horizon Europe, Award ID: 101071900. Gustavo Deco, AGAUR research support grant, Award ID: 2021 SGR 00917. Paul Klauser, Adrian and Simone Frutiger. Patric Hagmann, Swiss National Science Foundation, Award ID: 320030–197787/1.

## DATA AVAILABILITY STATEMENT

The code used for the analysis can be consulted in https://github.com/IraisGar/Perturbations-of-whole-brain-model-reveal-critical-areas-related-to-relapse-of-early-psychosis. The data used in this study will be available upon request; please contact I.G.M.L.

## Supplementary Material



## TECHNICAL TERMS

Functional connectivity (FC): Temporal correlation between regional fluctuations in cerebral blood flow in different areas of the brain.
Resting-state networks: Or intrinsic connectivity networks are areas of the brain that present synchronous activity.
Functional magnetic resonance imaging (fMRI): Type of MRI based on the registration of changes in blood flow to indicate which brain areas are more active in the moment of the scan.
Autoencoder (AE): Artificial neural network for unsupervised learning, it learns an efficient representation to encode and decode the data to and from a lower-dimensional space.
Diffusion tensor imaging: MRI modality that uses the random motion, magnitude and direction, of water to generate images of the neural pathways and connectivity.
Genetic algorithm: Adaptive heuristic search algorithms that use an evolutionary approach generating mutations in the current solution population with the aim to find solutions that better fit the chosen criteria.
Structural connectivity: Anatomical organization of the brain by means of fibre tracts.
Fully connected (FCN): Type of artificial neural network where the information between nodes flows such as every input neuron is connected to every neuron in the next layer.
t-distributed stochastic neighbor embedding or t-SNE: Statistical visualization method based on a stochastic neighbor embedding for dimensionality reduction.
Magnetization-prepared rapid acquisition gradient echo (MPRAGE): Whole brain imaging structural sequence that captures high tissue contrast with high spatial resolution and short scan time.
Diffusion spectrum imaging (DSI): Result of the use of specific MRI sequences processed with specialized software that generate images using the diffusion of the water molecules to generate contrast in MRI.
Hopf bifurcation: In mathematical theory of bifurcations it is a critical point where a system’s stability switches and a periodic solution appears.
