# Supplementary material for: Perturbations of whole-brain model reveal critical areas related to relapse of early psychosis
Source: Netw Neurosci. 2026 Jan 8;10(1):62–79. doi: 10.1162/NETN.a.502 (PMC12798648; doi:10.1162/NETN.a.502)
Supplement: Supplementary file 1 [file netn-10-1-62-s001.pdf]

COMPARISON MODELS

ACCURACY FOR THE DIFFERENT TESTED CONDITIONS

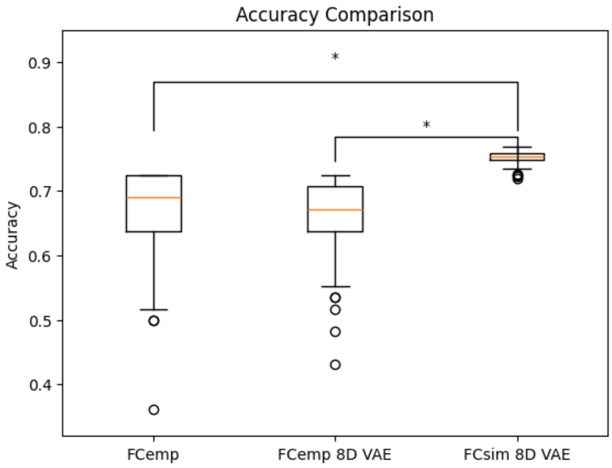

Supplementary Figure 1: Results of the classification accuracy of 100 FCNs when testing the classification of the 3 defined classes of psychotic cases using different conditions (from left to right): using the empiric FC matrices without 8D latent space encoding, using the empiric FC matrices with an 8D latent space encoding and using the simulated FC with an 8D latent space encoding. Significant differences in the distributions ($p < 0.05$) are indicated as *, as given by a Kolmogorov-Smirnov Test (KS Test).

T-SNE COMPARISON MODELS

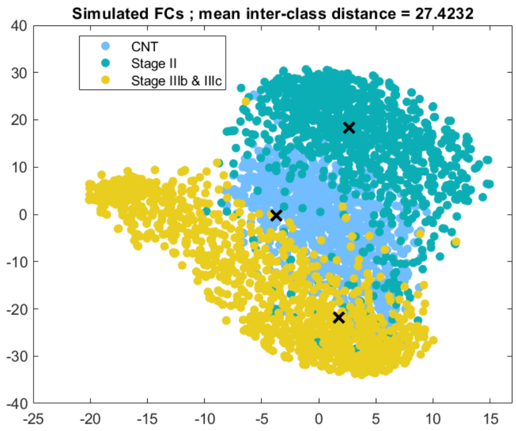

Supplementary Figure 2: *t-snr* 2D space trained with the simulated FC matrixes without them being encoded in an 8D latent space. Moreover, the centroids for each defined class were computed and the distance between all of them averaged to find a quantitative measure of the classes' separation in the low dimensional space.

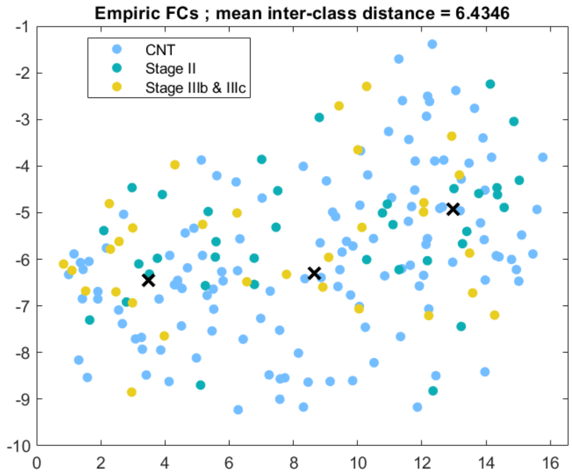

Supplementary Figure 3: *t-sne* 2D space trained with the empiric FC matrixes without them being encoded in an 8D latent space. Moreover, the centroids for each defined class were computed and the distance between all of them averaged to find a quantitative measure of the classes' separation in the low dimensional space.

NULL HYPOTHESIS

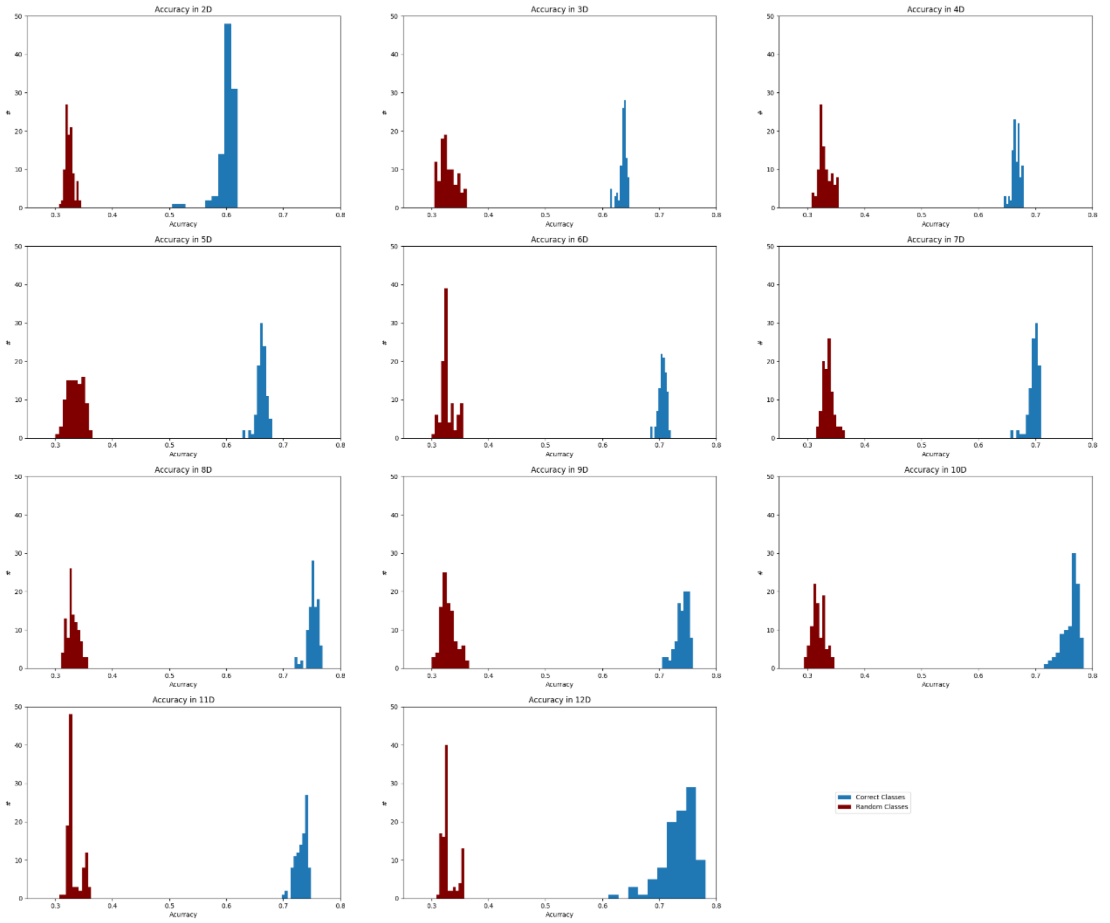

Supplementary Figure 4: Results of the classification accuracy of 100 FCNs when testing the classification of the 3 defined classes of psychotic cases using different number of latent dimensions during the training of the VAE models with, both, a correct labelling of the dataset (blue histogram) an incorrect labelling of the samples (red histogram).

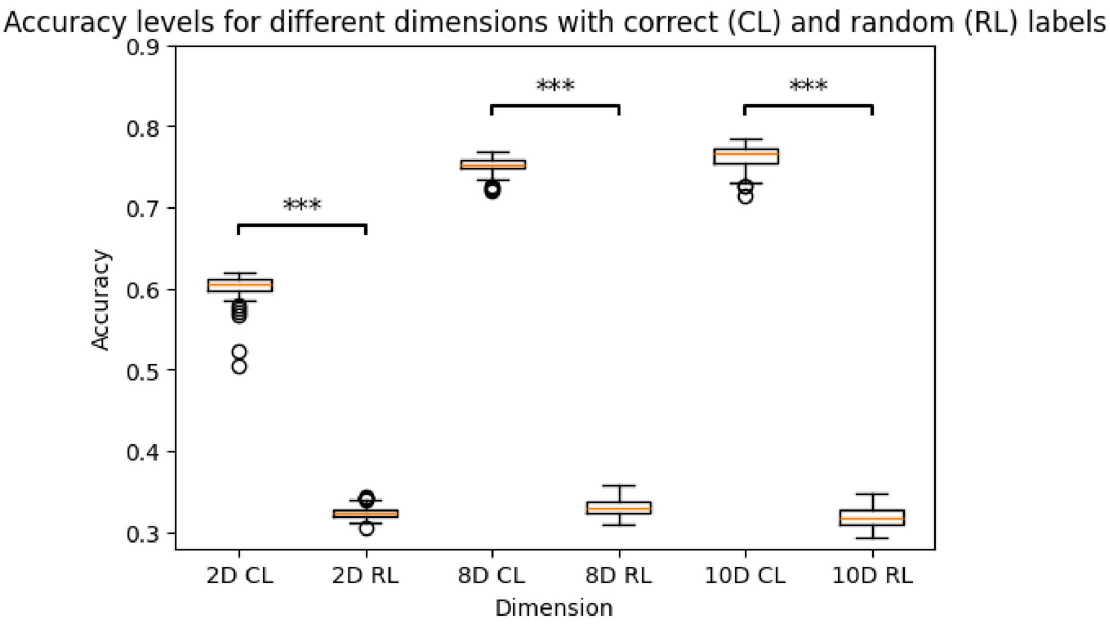

Supplementary Figure 5: Results of the classification accuracy of 100 FCNs when testing the classification of the 3 defined classes of psychotic cases using different number of latent dimensions during the training of the VAE models (from left to right): 2D latent space with correct labelling of the samples, 2D latent space with incorrect labelling of the samples, 8D latent space with correct labelling of the samples, 8D latent space with incorrect labelling of the samples, 10D latent space with correct labelling of the samples, 10D latent space with incorrect labelling of the samples. Significant differences in the distributions ($p < 0.01$) are indicated as ***, as given by a KS Test.

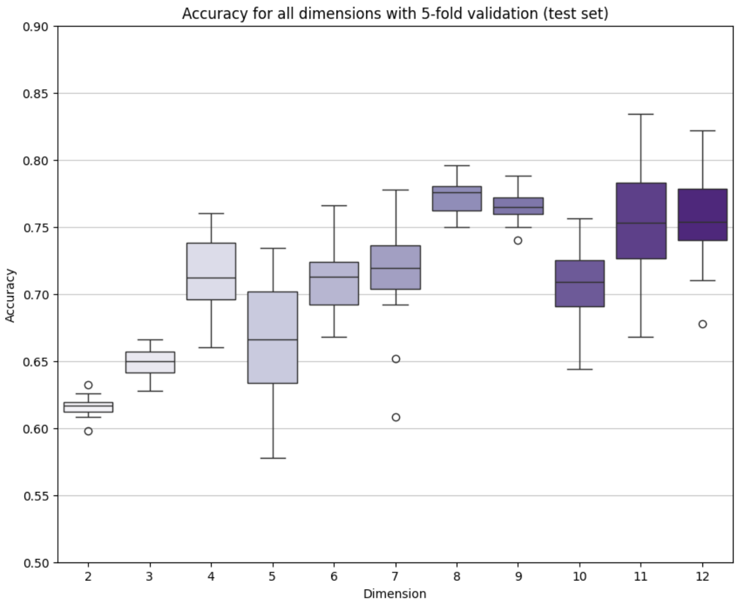

Supplementary Figure 6: Results of the classification accuracy (for an unseen test set) of a 5-fold cross validation for 100 FCNs when testing the classification of the 3 defined classes of psychotic cases using different number of latent dimensions during the training of the VAE models.

RESTING STATE NETWORKS

1	Visual	5	Limbic
2	Somatomotor	6	Frontoparietal
3	Attention (Dorsal)	7	Default
4	Attention (Ventral)	8	Subcortical

Supplementary Table 1: Resting State Networks definition.
